# Endotoxin- and ATP-neutralizing activity of alkaline phosphatase as a strategy to limit neuroinflammation

**DOI:** 10.1186/1742-2094-9-266

**Published:** 2012-12-11

**Authors:** Ruth Huizinga, Karim L Kreft, Sabina Onderwater, Joke G Boonstra, Ruud Brands, Rogier Q Hintzen, Jon D Laman

**Affiliations:** 1 Department of Immunology, Erasmus MC, University Medical Center, Dr. Molewaterplein 50, 3015 GE, Rotterdam, The Netherlands; 2 Neurology, Erasmus MC, University Medical Center Rotterdam, Rotterdam, The Netherlands; 3 Clinical Chemistry, Erasmus MC, University Medical Center Rotterdam, Rotterdam, The Netherlands; 4 MS Center ErasMS, Erasmus MC, University Medical Center Rotterdam, Rotterdam, The Netherlands; 5 Department of Biochemistry, Alloksys Life Sciences BV, Utrecht, The Netherlands

**Keywords:** Autoimmunity, Neuroimmunology, Lipopolysaccharide (LPS), Purinergic signalling, Multiple sclerosis

## Abstract

**Background:**

Alkaline phosphatase (AP) is a ubiquitously expressed enzyme which can neutralize endotoxin as well as adenosine triphosphate (ATP), an endogenous danger signal released during brain injury. In this study we assessed a potential therapeutic role for AP in inhibiting neuroinflammation using three complementary approaches.

**Methods:**

Mice were immunized to induce experimental autoimmune encephalomyelitis (EAE) and treated with AP for seven days during different phases of disease. In addition, serological assays to determine AP activity, endotoxin levels and endotoxin-reactive antibodies were performed in a cohort of multiple sclerosis (MS) patients and controls. Finally, the expression of AP and related enzymes CD39 and CD73 was investigated in brain tissue from MS patients and control subjects.

**Results:**

AP administration during the priming phase, but not during later stages, of EAE significantly reduced neurological signs. This was accompanied by reduced proliferation of splenocytes to the immunogen, myelin oligodendrocyte glycoprotein peptide. In MS patients, AP activity and isoenzyme distribution were similar to controls. Although endotoxin-reactive IgM was reduced in primary-progressive MS patients, plasma endotoxin levels were not different between groups. Finally, unlike AP and CD73, CD39 was highly upregulated on microglia in white matter lesions of patients with MS.

**Conclusions:**

Our findings demonstrate that: 1) pre-symptomatic AP treatment reduces neurological signs of EAE; 2) MS patients do not have altered circulating levels of AP or endotoxin; and 3) the expression of the AP-like enzyme CD39 is increased on microglia in white matter lesions of MS patients.

## Background

Inflammatory and neurodegenerative responses in the central nervous system (CNS) are strongly affected by infections that occur in the periphery [1]. This is clinically illustrated by the fact that infections are an important risk factor for the development of relapses in multiple sclerosis (MS) [2,3]. In addition, infections may enhance the severity and/or duration of clinical exacerbations. Evidence from experimental models also demonstrates that systemic administration of bacterial compounds such as LPS or staphylococcal enterotoxin induces clinical relapses in mice recovered from experimental autoimmune encephalomyelitis (EAE) [4,5]. Infections may cause activation of autoreactive lymphocytes in the periphery in a non-specific manner [5,6]. Alternatively, bacterial compounds when released into the bloodstream may increase blood–brain barrier permeability [7], subsequently activating microglia, which secrete proinflammatory cytokines promoting neurodegeneration [8]. Recent studies further demonstrate that manipulation of the normal gut microbiota by antibiotics, germ-free conditions and administration of *Bacteroides fragilis* polysaccharides affects EAE incidence and severity [9-11]. It is conceivable that both exogenous (LPS) and endogenous phosphorylated compounds such as adenosine triphosphate (ATP) at least partly mediate these effects. Together, these findings suggest that patients with MS may benefit from an early control of infections and neutralization of microbial compounds of normal gut flora.

A promising strategy for the neutralization of bacterial endotoxin and pro-inflammatory extracellular nucleotides is treatment with alkaline phosphatase (AP), an enzyme ubiquitously expressed in mammalian tissues and present in body fluids. AP hydrolyzes the diphosphoryl lipid A moiety of LPS, generating the non-toxic monophosphoryl lipid A [12]. Endogenous AP plays a role in the defense against Gram-negative bacteria [13] and is pivotal for normal gut homeostasis [14,15]. AP has beneficial effects in several animal models of inflammatory diseases, including sepsis, inflammatory bowel disease and colitis [16-18].

In addition to detoxification of exogenous compounds, such as LPS and bacterial CpG [19], AP also deactivates endogenous molecules such as ATP, which serves as an immunological danger signal when present at high concentrations (>100 μM) in the extracellular space [20]. ATP, produced by bacteria and released in large concentrations from damaged cells, is sensed by purinergic P2 receptors [21]. In the intestinal lamina propria, ATP is critical for the differentiation of Th17 cells [22].

CD39 and CD73 are two other enzymes that are involved in ATP metabolism, thus having overlapping functions with AP. CD39, like AP, mediates the conversion of ATP via ADP to AMP. CD73 and AP both convert AMP into adenosine. The two enzymes are expressed by regulatory T cells (Treg) and are crucial for the immunosuppressive function of Treg by decreasing local ATP concentrations and increasing the immunosuppressive adenosine [23]. In addition, CD73 expression and adenosine signalling is pivotal for leukocyte entry into the CNS of mice with EAE [24].

Because infections often precede MS relapses and given that AP detoxifies endogenous and exogenous innate activating signals, we hypothesize that AP has a beneficial role in limiting neuroinflammation in MS. In this study we therefore aimed to: 1) determine the prophylactic and therapeutic potential of AP in EAE, a mouse model of MS; 2) determine plasma AP levels in MS patients in relation to endotoxin exposure; and 3) assess the expression and cellular sources of AP in relation to CD39 and CD73 in MS brain tissue.

## Methods

### EAE induction and AP treatment

Ten-week old female C57BL/6 mice (Harlan) were immunized with 50 μg MOG35-55 peptide (Peplogic, London, UK) emulsified in complete Freund’s adjuvant (CFA; Difco Laboratories, Detroit, MI, USA). Animals were injected s.c. with a total of 200 μl adjuvant divided over four ventral sites in the axillary and inguinal regions. Pertussis toxin (100 ng/mouse; Sigma-Aldrich, Zwijndrecht, The Netherlands) was given i.p. on day 0 and 2. AP (Biozyme Laboratories, Blaenavon, Gwent, UK) was injected i.p. (5 U/mouse/day) during the different phases of EAE, that is, the priming phase (day 0 to 6), the onset of clinical signs (day 7 to 13) or the plateau phase (day 14 to 20). Bovine intestinal AP (Biozyme Laboratories, Gwent, UK) or control diluent (50% glycerol, 5 mM Tris, 5 mM MgCl_2_ 0.1 mM ZnCl_2_ at pH 7.0) were diluted prior to use in 0.9% NaCl. Mice were treated for a maximum of seven days in order to prevent the occurrence of serum sickness. Mice were weighed and scored for clinical signs daily as follows: 0, no disease; 1, tail paralysis; 2, paraparesis; 3, partial limb paralysis; 4, complete limb paralysis; 5, moribund. Animals exhibiting signs that were less severe than typically observed for the standard score were scored 0.5 less than the indicated grade. The scoring was performed by investigators who were blinded to treatment assignment. The animal experiments were approved by the animal ethical committee and performed according to local and national guidelines for animal experimentation. The group sizes (n = 10) were calculated by power analysis in order to reach significance with an EAE score difference of one grade using a standard deviation of 0.8, power of 80% and a significance level of 0.05.

### T-cell proliferation assay

Spleens were isolated on day 28 after EAE induction. A single-cell suspension was prepared and erythrocytes were lysed by incubating with Gey’s reagent for 5 minutes. Cells were washed and seeded at 4 × 10^5^ cells/well in Iscove’s modified Dulbecco’s medium (IMDM) supplemented with 2% normal mouse serum, 100 U/ml penicillin, 100 μg/ml streptomycin and 2 mM glutamine (all from Lonza, BioWittaker, Verviers, Belgium). MOG35-55 peptide was added at 0.1, 1 or 10 μg/ml and phytohemagglutinin (PHA) was used at 10 μg/ml as positive control. After three days, [^3^H]-thymidine was added at 0.5 μCi/well for the last 18 hours. Proliferation was measured using a β-counter (Wallac MicroBeta, PerkinElmer, Waltham, MA, USA) and expressed as mean counts per minute (cpm).

### ELISA

Supernatants of mouse splenocyte cultures were collected on day three after *in vitro* stimulation and assessed for cytokine content using ELISA kits for IFN-γ, tumor necrosis factor α (TNF-α) (both from BD Biosciences, Breda, The Netherlands) and IL-17A (R&D, Abingdon, UK).

### Patients and controls

Patients (n = 26; detailed in Table 1) were selected based on a diagnosis of clinically definite MS according to the McDonald criteria [25]. Patients were not treated with immunomodulatory compounds at the time of blood sampling. The control groups consisted of healthy subjects (n = 18; anonymized laboratory co-workers) and patients with other non-inflammatory and non-infectious neurological diseases (n = 11). Sera of patients and controls were collected at the outpatient clinic of Neurology at the Erasmus MC, and stored at −80°C until use. The study was approved by the Medical Ethical Committee of the Erasmus MC and written informed consent was obtained from patients and controls who participated in this study. 

**Table 1:** Clinical characteristics of patients and controls

	**HC**	**MS**	**OND**
	**(number = 18)**	**(number = 26)**	**(number = 11)**
Age at onset (SD)	NA	37 (12)	NA
Age at sampling (SD; range)	37 (12; 21 to 59)	Total MS: 41 (12; 19 to 59)	39 (13; 20 to 57)
		RR-MS: 34 (11; 19 to 59)	
		PP-MS: 46 (9; 31 to 58)	
Female (%)	78	73	45
Disease duration, years (SD)	NA	3 (1)^a^	NA
Presenting symptoms (number)			
Optic nerve		2	
Spinal cord	NA	16	NA
Brainstem/cerebellum		3	
Cerebrum		5	
Raised IgG-index or oligoclonal bands (number)	NA	18/23	NA

^a^At sampling, five patients were still clinically isolated syndrome (CIS) patients. Of these patients, four developed RR-MS after sampling. One CIS patient was at high risk for developing MS (fulfilment of the Barkhof criteria on MRI and oligoclonal bands and raised IgG-index in the CSF) and was followed for seven years without new neurological complaints. CSF, cerebrospinal fluid; HC, healthy controls; IgG, immunoglobulin G; MRI, magnetic resonance imaging; MS, multiple sclerosis; OND, other neurological disease; PP-MS, primary progressive MS; RR-MS, relapsing-remitting MS; SD, standard deviation.

^a^At sampling, five patients were still clinically isolated syndrome (CIS) patients. Of these patients, four developed RR-MS after sampling. One CIS patient was at high risk for developing MS (fulfilment of the Barkhof criteria on MRI and oligoclonal bands and raised IgG-index in the CSF) and was followed for seven years without new neurological complaints. CSF, cerebrospinal fluid; HC, healthy controls; IgG, immunoglobulin G; MRI, magnetic resonance imaging; MS, multiple sclerosis; OND, other neurological disease; PP-MS, primary progressive MS; RR-MS, relapsing-remitting MS; SD, standard deviation.

### Serological assays

Total AP activity was measured by a routine enzymatic assay according to the recommendations of the International Federation of Clinical Chemistry and using a Cobas 6000 automated analyzer (Roche Diagnostics, Almere, The Netherlands). Liver, bone, intestine, placenta and bile AP iso-enzymes were separated by agarose gel electrophoresis using wheat germ agglutinin. Bands were quantified using densitometry. All measurements were performed in the accredited Department of Clinical Chemistry of the Erasmus MC by qualified personnel blinded to sample identity. Plasma endotoxin levels were measured using the chromogenic Limulus Amebocyte Lysate (LAL) endpoint assay (Lonza), according to the manufacturers’ procedures. The assay was strictly controlled by a series of practical measures including the use of automatic pipets to minimize time differences between wells. Endotoxin-reactive IgM and IgG antibodies in sera of MS patients and control subjects were measured using the EndoCab ELISA (Hycult Biotechnology, Uden, The Netherlands).

### Enzyme histochemistry and immunohistochemistry

Post-mortem brain material of five prototypical patients with clinically definite MS and four non-demented controls (Table 2) was obtained from The Netherlands Brain Bank, Netherlands Institute for Neuroscience, Amsterdam. All material was collected from donors from whom written informed consent for brain autopsy and the use of the material and clinical information for research purposes had been obtained by the Netherlands Brain Bank. 

**Table 2:** Clinical characteristics of patients included for postmortem studies

**Patient**	**Gender**	**Age at death (years)**	**Disease duration (years)**	**MS disease form**	**Cause of death**
NDC1	Male	73	NA	NA	Colon carcinoma with liver metastases
NDC2	Female	90	NA	NA	Unknown
NDC3	Female	68	NA	NA	Metastasized mamma carcinoma
NDC4	Male	84	NA	NA	Heart failure by uremia
MS1	Female	41	11	SP	Natural death
MS2	Female	50	17	Chronic progressive	Euthanasia
MS3	Female	55	21	SP	Possible CVA
MS4	Male	64	34	PP	End-stage progressive MS
MS5	Female	76	34	SP	Respiratory insufficiency of unknown origin

CVA, cerebrovascular accident; MS, multiple sclerosis; NA, not applicable; PP, primary progressive; SP, secondary progressive.

CVA, cerebrovascular accident; MS, multiple sclerosis; NA, not applicable; PP, primary progressive; SP, secondary progressive.

Frozen sections were fixed with acetone/0.05% H_2_O_2_ for 10 minutes and stained for endogenous AP activity using naphthol-AS-MX-phosphate and Fast Blue BB base (Sigma-Aldrich), resulting in a blue precipitate. Sections were subsequently incubated with 10% normal goat serum and 5% normal human serum in PBS/0.1% BSA for 30 minutes. The primary antibodies anti-CD39 (clone BU61; Ancell Corporation, Bayport, MN, USA), anti-CD73 (clone 4G4; Hycult Biotechnology), anti-MOG (18-18-C5) and anti-HLA-DR (clone L243; BD Biosciences) were allowed to bind overnight at 4°C. After washing, sections were incubated with biotinylated goat anti-mouse IgG1 (Southern Biotechnology Associates, Birmingham, AL, USA) in PBS/1% BSA/1% normal human serum for 45 minutes at room temperature and with avidin-biotin complexes (Vector Laboratories, Peterborough, UK) for 30 minutes. For double staining with HLA-DR, sections were incubated with alkaline phosphatase-conjugated goat anti-mouse IgG2a. Bound complexes were visualized with 3-amino-9-ethylcarbazole (AEC) resulting in a translucent red product. HLA-DR in double stainings was revealed using naphthol-AS-MX-phosphate and Fast Blue BB base in the presence of 1.5 mM levamisole (Sigma-Aldrich), to inhibit endogenous AP. Aspecific binding was evaluated by replacing primary antibodies with isotype-matched control antibodies.

### Statistical analysis

Data were analyzed using Graphpad prism software or SPSS using parametric or non-parametric tests as appropriate and *P* <0.05 was considered statistically significant.

## Results

### Pre-symptomatic treatment with alkaline phosphatase reduces EAE severity

To investigate whether AP modulates the EAE disease course, we induced chronic EAE in C57BL/6 mice using a group size of 9 to 10 animals per group and treated mice with AP at different stages of disease, that is, during the priming phase (day 0 to 6), the onset of clinical signs (day 7 to 13) or during the plateau phase (day 14 to 20). Mice were treated with 5 U AP/day (approximately 100x baseline level of AP) which is similar to other experimental studies.

**Figure 1:**
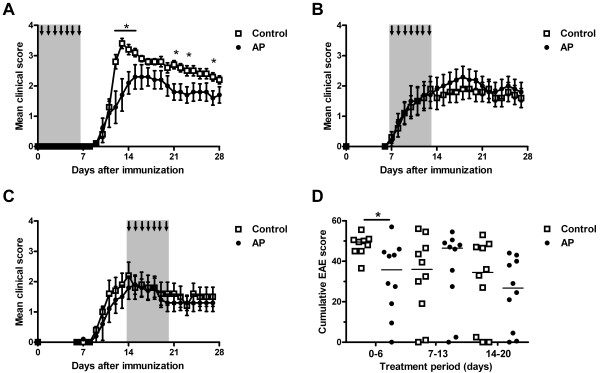
**Pre-symptomatic treatment with AP reduces EAE severity.** C57BL/6 mice were immunized with 50 μg MOG35-55 in CFA and treated daily with 5 U AP or vehicle control from day 0 to 6 as indicated by arrows and shading (**A**, n = 9 control group; n = 10 AP-treated group), day 7 to 13 (**B**, n = 10) or day 14 to 20 (**C**, n = 10). Only mice treated early after immunization showed reduced clinical signs for EAE as compared to vehicle-treated mice. Shown are mean clinical scores ± sem. The cumulative EAE score in the pre-symptomatic treatment group was lower in AP-treated animals compared to controls (**D**, line indicates median). * *P* <0.05 Mann–Whitney U test. AP, alkaline phosphatase; CFA, complete Freund’s adjuvant; EAE, experimental autoimmume encephalomyelitis; sem, standard error of the mean.

AP treatment of MOG35-55-immunized mice during the priming phase did not affect disease incidence, which was 100% in the control group and 90% in the AP-treated group. Also, the onset of clinical signs was comparable between the groups, 10.7 ± 0.2 days in the control group versus 12.2 ± 0.9 days in the AP-treated group (*P* = 0.27, Mann–Whitney U test). However, animals treated with AP experienced less severe signs of EAE which was most pronounced at the peak of the disease, that is, day 12 to 14 post-immunization, as reflected by a significant decrease in mean clinical score at day 13 from 3.4 ± 0.2 in the control group to 1.7 ± 0.5 in the AP-treated group (*P* = 0.029, Mann–Whitney U test; Figure 1A). Accordingly, the cumulative EAE score was significantly reduced in AP-treated animals as compared to vehicle-treated animals (*P* = 0.016, Mann Whitney U test; Figure 1D). When animals were treated at later time points, that is, from day 7 to day 13 or from day 14 to day 20, no differences were observed in clinical EAE scores (Figure 1B-D). 

### Treatment with alkaline phosphatase reduces T-cell proliferation to MOG35-55

**Figure 2:**
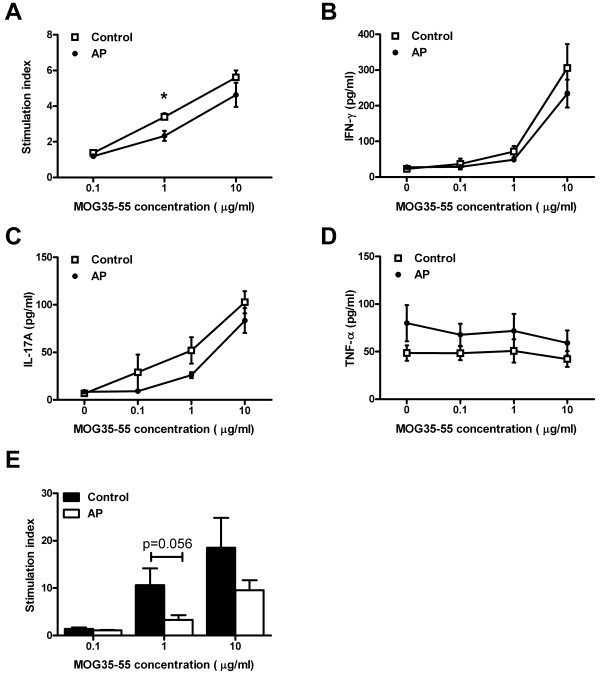
**Early treatment with AP reduces T-cell proliferation to MOG35-55.** C57BL/6 mice were immunized with MOG35-55 in CFA and treated daily with AP from day 0 to 6. Splenocytes were isolated at day 28 and proliferation of 9 individual mice per group was measured by [^3^H]-thymidine incorporation in response to increasing doses of MOG35-55 (**A**). The supernatant of these cultures was assessed for the presence of IFN-γ (**B**), IL-17A (**C**) and TNF-α (**D**). In a separate experiment, mice (n = 5 per group) were treated with AP or control diluent from day 0 to 6, splenocytes were harvested on day 10 and proliferation to MOG35-55 was assessed as described above (**E**). Data indicate mean ± sem; * *P* <0.05 Mann–Whitney U test. AP, alkaline phosphatase; CFA, complete Freund’s adjuvant; sem, standard error of the mean; TNF-α, tumor necrosis factor α.

To determine whether mice with reduced clinical signs due to early AP treatment showed decreased immune responses to the immunogen MOG35-55, splenocytes were isolated at 28 days after immunization and assessed for proliferative capacity by the [^3^H]-thymidine incorporation assay (nine mice per group). No difference was observed in basal splenocyte proliferation (680 ± 150 for the control group and 870 ± 193 for AP-treated animals) or in PHA-induced proliferation (1,923 ± 445 for the control group and 2,121 ± 330 for AP-treated animals) although it must be noted that the peak of PHA-induced proliferation is already after one day of culture. However, mice treated with AP had a modest but significant reduction in antigen-specific proliferation. Splenocytes from control animals cultured in the presence of 1 μg/ml MOG35-55 showed a stimulation index (SI) of 3.4 ± 0.2 while splenocytes from AP-treated animals had a stimulation index of 2.3 ± 0.3 (*P* <0.017; Mann Whitney U test; Figure 2A). Splenocytes cultured *in vitro* produced IFN-γ and IL-17A in response to MOG35-55 (Figure 2B and C). No significant differences in cytokine production were observed between cells from AP-treated and vehicle-treated animals, although a consistently lower level of IL-17A was produced by splenocytes from AP-treated mice (*P* = 0.11, Mann–Whitney U test). Production of TNF-α was higher in AP-treated animals, although not significantly, and was not further upregulated in response to MOG35-55 (Figure 2D). 

To investigate whether AP treatment affects the priming of auto-reactive T cells, we performed a separate animal experiment (5 mice per group) and harvested splenocytes on day 10, before onset of EAE. There was a trend (*P* = 0.056) towards decreased proliferation in the AP-treated group (Figure 2E). Consistent with the results obtained at day 28, the strongest effect of AP treatment was observed at a concentration of 1 μg/ml MOG35-55.

The histopathology of the spinal cord was assessed 28 days after immunization in mice treated pre-symptomatically with AP or control diluent. All mice, irrespective of treatment, showed characteristic EAE lesions in the spinal cord, consisting of activated F4/80^+^ macrophages, CD3^+^ T cells and B220^+^ B cells. General microscopic examination showed no striking differences in the number or size of lesions between the groups.

In summary, we demonstrate that only pre-symptomatic AP treatment reduces clinical signs of EAE and that splenocytes of AP-treated mice show reduced proliferation in response to MOG35-55 with no differences in cytokine profile.

### AP in relation to endotoxin exposure in MS and controls

Since AP is important for detoxifying endotoxin, we determined the total AP activity, the presence of AP isoforms, as well as the endotoxin levels in the plasma of 26 patients with MS and 29 controls. Moreover, correlations between total AP activity and endotoxin levels were examined.

**Figure 3:**
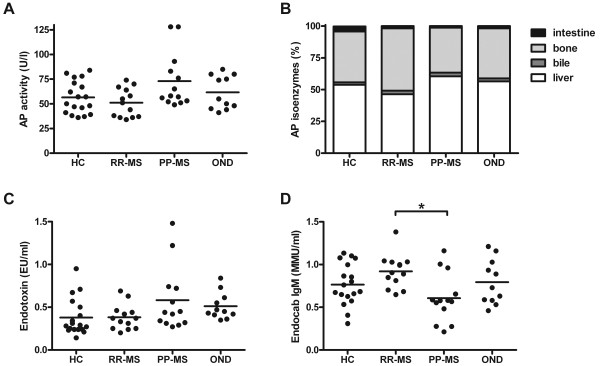
**AP activity and endotoxin-reactive antibodies in MS and control subjects.** Plasma of primary progressive MS (PP-MS) patients, relapsing-remitting MS (RR-MS), healthy controls (HC) and other neurological disease (OND) controls were assessed for total AP activity (**A**), the four AP isoenzymes (**B**) and endotoxin levels (**C**). PP-MS patients showed a decreased level of endotoxin-specific IgM (**D**; * *P* <0.05, Bonferroni-corrected Mann–Whitney U test). AP, alkaline phosphatase; IgM, immunoglobulin M; MS, multiple sclerosis.

Although a higher AP activity was noted in the plasma of PP-MS patients (73.0 ± 7.7 U/l) compared to RR-MS patients (51.1 ± 4.0 U/l; Figure 3A), subgroup analysis demonstrated no significant differences (*P* = 0.075; Kruskal-Wallis test). Similarly, the relative level of AP isoforms was comparable between the different subgroups (Figure 3B). Within the group of all MS patients there was a significant correlation between age and total AP levels (r_s_ = 0.407; *P* = 0.039; n = 26) which could account for the observed differences in AP activity between MS subgroups. 

The endotoxin exposure was measured both directly using a LAL assay and indirectly by determining endotoxin core antibodies (Endocab). Endotoxin levels did not differ between MS patients and control subjects, or between RR-MS and PP-MS patients (Figure 3C). A significant difference in the level of Endocab IgM was, however, found between RR-MS and PP-MS patients (*P* = 0.024, Bonferroni-corrected Mann–Whitney U test; Figure 3D). Age was significantly correlated with Endocab IgM levels (r_s_ = −0.39; *P* = 0.003; n = 55), which may account for the observed differences between RR-MS patients and PP-MS patients. No differences were found in Endocab IgG levels. Endotoxin levels did not correlate with Endocab IgM (r_s_ = −0.029; *P* = 0.83; n = 55). A correlation between AP activity and endotoxin levels was found in healthy controls (r_s_ = 0.52; *P* = 0.027) but not in MS patients (r_s_ = 0.036; *P* = 0.86).

### Differential expression of detoxifying enzymes in MS lesions

Finally, we determined the activity of endogenous AP in MS patients (n = 5) and non-demented control brain tissue (n = 4) using enzyme histochemistry revealing AP action in acetone-fixed frozen sections. Additionally, the expression of CD39 and CD73 was determined, as these molecules have nucleotidase activity similar to AP [30].

**Figure 4:**
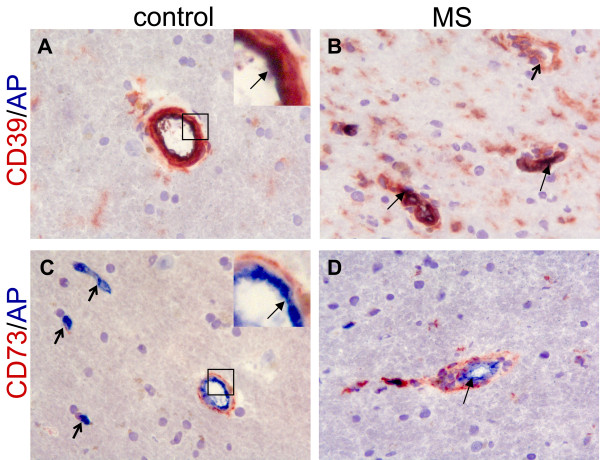
**Expression of detoxifying enzymes on blood vessels in MS and control brain tissue.** CD39 (red) was expressed on blood vessels in brain tissues of both non-demented controls (**A**) and MS patients (**B**), partly together with AP (blue) at the endothelial lining (resulting in a purple/black precipitate indicated by the arrow in the inset in A). CD39 was also expressed in the parenchyma on cells with a ramified morphology, most notably in MS tissue. A CD39 single positive blood vessel is indicated by the open arrow (B). CD73 (red) was also found on blood vessels from non-demented controls (**C**) and MS patients (**D**), but not on endothelial cells that expressed AP (bright blue, arrow in inset). Instead CD73 reactivity was observed at the border of the Virchow-Robin space, the glia limitans. Open arrows in C indicate AP single positive blood vessels (bright blue). Magnification 250x. AP, alkaline phosphatase; MS, multiple sclerosis.

Consistent with its known expression pattern on blood vessels in the periphery, AP was evident in endothelial cells of blood vessels (Figure 4). CD39 was also expressed on blood vessels, similar to AP, both in control and MS tissue (Figure 4A and B). CD39 expression on blood vessels was less restricted than AP, as it was found on both endothelial cells and within the smooth muscle layer (Figure 4A and B). In contrast to AP, CD73 was not present on cerebral endothelium, but was more pronounced at the border of the Virchow-Robin space, possibly reflecting the glia limitans. CD73 expression on blood vessels was comparable between MS and control subjects (Figure 4C and D). 

**Figure 5:**
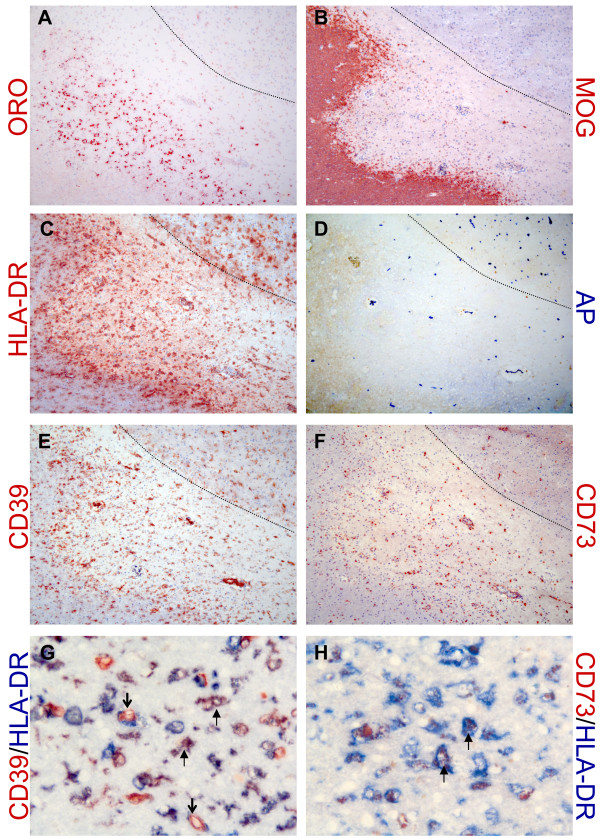
**Differential expression of detoxifying enzymes in MS lesions.** AP, CD39 and CD73 expression is shown in a chronic active MS lesion, positive for oil red O (**A**), lacking reactivity for myelin oligodendrocyte glycoprotein (**B**) and containing many HLA-DR^+^ cells (**C**). The grey matter (border is indicated by dashed line in panels A-**D**) contains many AP^+^ blood vessels. CD39 expression is prominent especially at the lesion border (**E**) while CD73 (**F**) is present throughout the lesion. CD39 was expressed on cells with a ramified morphology that co-expressed HLA-DR (resulting in purple staining indicated by arrows; **G**). Open arrows indicate CD39 single-positive cells. CD73 (red) was present as punctate staining in the lesion inside HLA-DR^+^ cells (blue; **H**). Note that D, G and H lack hematoxylin counterstaining. Magnification 100x (A-F) and 250x (G,H). AP, alkaline phosphatase; MS, multiple sclerosis.

In MS white matter lesions with active demyelination and inflammation (Figure 5A-C), AP was also expressed on blood vessels (Figure 5D) and was similar to that of normal-appearing white matter or control brain tissue. In contrast, an increased expression of CD39 and CD73 was observed (Figure 5E and F). CD39 was evident on cells with a ramified morphology that co-expressed HLA-DR representing microglia/macrophages (Figure 5G). Of note, some HLA-DR^+^ cells, especially those located adjacent to capillaries and in the center of the lesion, did not express CD39. Within actively demyelinating MS lesions, a punctate staining of CD73 was noted and double labeling with HLA-DR showed that CD73-positive staining was localized within HLA-DR-positive cells (Figure 5H). 

## Discussion

MS exacerbations are often associated with preceding infections [2,3]. Moreover, recent experimental studies demonstrate that exposure to LPS or the commensal microbiota is necessary for the development of neuroinflammatory disease in mice with myelin-reactive T cells [5,11]. These findings strongly suggest that microbes play a crucial role in the initiation and propagation of inflammatory responses that mediate CNS pathology. This may occur by activation of autoreactive T cells in the periphery [5] or through activation of microglia that initiate inflammatory responses in the brain parenchyma [31]. In the latter scenario, systemic inflammation leads to a phenotypic switch of microglia, from anti-inflammatory to proinflammatory [8,31]. Importantly, preactive MS lesions, characterized by activated microglia, do not always develop into demyelinating lesions [32] and it is tempting to speculate that systemic infections could act as a driving force of the development of demyelinating lesions. Irrespective of the mechanism, it can be argued that MS patients may benefit from neutralization of microbial compounds and endogenous danger signals as an attempt to prevent subsequent propagation of inflammatory responses in the CNS. In this study we hypothesized a protective role for AP through its potent detoxifying activity of LPS and endogenous danger signals, such as ATP. We demonstrate that pre-symptomatic AP administration to animals in which autoimmunity to MOG peptide was induced, resulted in the reduction of clinical signs of EAE.

**Figure 6:**
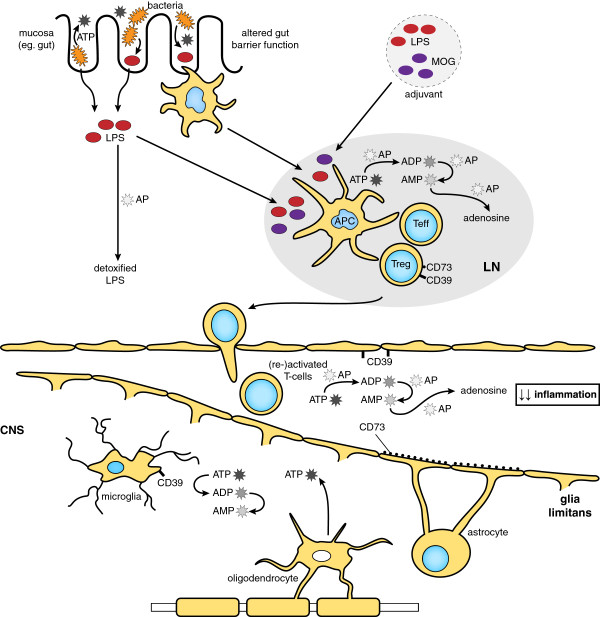
**Proposed model for the beneficial role of AP, CD73 and CD39 in the generation of autoreactive T cells and subsequent neuroinflammation.** AP detoxifies LPS derived from the gut microbiota or from the adjuvant in the case of EAE. During activation of T cells in the peripheral lymph nodes (LN) ATP is generated. CD73 and CD39, expressed on regulatory T cells, may reduce the high ATP concentration, which serves as an immunological danger signal. CD39 and AP convert ATP into ADP and AMP, whereas CD73 and AP convert AMP into the immunosuppressive adenosine. In the CNS, CD39 and CD73 are present on the endothelium and the glia limitans, respectively, where they may also reduce extracellular ATP levels. Within the lesion, stressed oligodendrocytes release ATP, which can be converted by CD39 upregulated on activated microglia. AP, alkaline phosphatase; CNS, central nervous system; EAE, experimental autoimmune encephalomyelitis; LPS, lipopolysaccharide.

There are several mechanisms that could explain how AP treatment resulted in a reduction of clinical severity in EAE (Figure 6). First, AP may neutralize the LPS present in the adjuvant and needed for the priming of pathogenic MOG-reactive T cells. In experimental colitis induced with dextran sodium sulfate, the LPS-neutralizing activity of AP is regarded as the principal mode of action [17]. Second, AP may detoxify endogenous substrates, such as ATP, and hence interfere with CD4^+^ T cell activation, as ATP was recently shown to provide autocrine signals for activated T cells [33]. Our finding that splenocytes of AP-treated mice proliferated less efficiently to MOG-peptide is in support of both mechanisms. The overall proliferation capacity of splenocytes was not affected by *in vivo* AP treatment; however, it must be noted that the proliferation to PHA was measured after three days and not at the optimum of one day. 

AP treatment may also act to limit inflammatory responses in the brain. ATP has been shown to mediate the microglial response to local brain injury [34] and has excitotoxic effects on oligodendrocytes [35]. We did not find a beneficial role for AP treatment (via the i.p. route) during the acute or chronic phase of disease. However, it is still possible that local administration of AP can interfere with microglial activation and oligodendrocyte death. The lack of clinical improvement after AP administration during the acute or chronic phase of disease may also be explained by the fact that the plasma residence time of the applied bovine AP is relatively short (in the order of minutes to a maximum of two hours [16]). Thus, ongoing inflammation is only in-part and transiently modulated. In contrast, the neutralization of endotoxin and ATP by the short-acting AP may have more impact at the onset of inflammation, as observed in experimental models of inflammatory bowel disease [18].

The treatment efficacy of AP in EAE was moderate, which is consistent with previous reports on AP therapy in other experimental immune mediated disorders (summarized in Table 3). Our finding that only early treatment with AP was able to decrease the clinical severity of EAE may suggest that the therapeutic potential of AP for patients with MS remains limited to preventing the priming of new T-cell responses. Short-term AP treatment may be considered when there is a high risk for a clinical relapse, for example during infections or when there is evidence of gut barrier dysfunction that leads to systemic release of microbial compounds (translocation). Unlike antibiotics, AP is able to detoxify these microbial compounds. Likely an AP isoenzyme with more prolonged plasma activity than the applied bovine intestinal AP (that is, placental AP) will perform better in this respect. 

**Table 3:** Overview of studies demonstrating a detoxifying role of AP *in vivo*

**Model**	**Duration of model**	**Inducing agent or adjuvant**	**Treatment regime**	**Result**	**Reference**
Acute myocardial infarction in BALB/c mice	24 hours	None (coronary artery ligation)	BIAP, 5 U i.v. prophylactic	Reduction of IL-6 and MCP-1 by 40%, IL-1β reduced by 30%. No effect on IL-10	Fiechter et al., submitted
Colitis in C57BL/6 mice	12 days	Dextran sulphate sodium (DSS)	BIAP orally, 100 U/day starting four days after disease induction	Reduction in body weight loss and TNF-α. Reduced gut leukocyte infiltration and tissue damage	[18]
Sepsis and lethal *E. coli* infection in BALB/c mice	24 hours	Live *E. coli* i.p.	Human placental AP, 1.5 U i.v.	Reduction in mortality.	[26]
LPS toxicity in mice and piglets	24 and 72 hours	Live *E. coli* i.p. in mice or LPS i.v. in piglets (200 ng/kg bodyweight)	BIAP i.v., 1.5 U in mice or 3.000 U in piglets	Increased survival in mice from 20% to 80%. Reduction in TNF-α by 98% in piglets. No toxicity of BIAP 4000 U/day for 28 days	[16]
Liver ischemia-reperfusion in rats	24 hours	None (clamping of hepatic blood vessels)	BIAP single dose i.v. 0.5 U/g bodyweight	Decreased neutrophil influx and tissue damage	[27]
Secondary peritonitis in C57BL/6 mice	72 hours	Endogenous gut microbiota (due to cecal ligation puncture)	BIAP single dose 0.15 U/g bodyweight	Reduced inflammation and hepatocellular and pulmonary damage	[28]
Septic shock in sheep	30 hours	Feces injection i.p.	BIAP bolus 60 U/kg and continuous infusion 20 U/kg/h for 15h	Reduced IL-6, improved gas exchange and longer survival	[29]
MOG35-55 induced EAE in C57BL/6 mice	28 days	Complete Freund’s adjuvant and pertussis toxin	Presymptomatic BIAP, 5 U/day i.p. for 7 days	Reduced clinical signs, reduced T-cell proliferation to immunogen	Huizinga et al. (this study)

BIAP, bovine intestinal alkaline phosphatase. LPS, lipopolysaccharide; TNFα, tumor necrosis factor α.

The efficacy of AP has been studied in several human inflammatory diseases and conditions. AP treatment was safe [36,37] and was associated with short-term clinical improvement and reduction of C-reactive protein in ulcerative colitis [38]. AP treatment also resulted in more prominent recovery of creatinine clearance in patients with sepsis-induced acute kidney injury [37]. The safety and efficacy of short-term AP treatment is currently under investigation for acute rheumatoid arthritis (ClinicalTrials.gov Identifier: NCT01416493). Animal models in which AP was shown to be effective were mostly acute conditions, including septic shock, acute myocardial infarction and peritonitis (summarized in Table 3). However, beneficial effects have also been reported in chronic colitis [17,39].

We also determined the expression of AP and its related enzymes CD39 and CD73 in MS brain tissue. The observed AP expression pattern was largely similar to that in rodents and primates as reported previously [40,41]. In contrast to Alzheimer’s disease [42], we did not find increased AP expression in MS brain compared to controls. It is possible that enzyme histochemistry as used in this study is less sensitive in detecting differences than enzyme assays of whole tissue homogenates. CD39 was predominantly expressed on blood vessel endothelium, as was AP, and on microglia, which is consistent with an earlier study [40]. In white matter lesions of MS patients, CD39 expression was also found on microglia. CD39 may play a role in the microglial response to inflammation and tissue damage as it converts extracellular toxic ATP, which is released upon tissue damage, to ADP and AMP. Not all HLA-DR^+^ cells co-expressed CD39, especially those that surrounded capillaries and some cells in the center of the lesion, suggesting that CD39 is differentially expressed by (perivascular) macrophages and microglia. In the periphery, CD39 is also expressed by Treg cells [43]. CD39^+^ Treg cells were recently shown to suppress pathogenic Th17 cells and were reduced in peripheral blood of MS patients [44].

In contrast to AP and CD39, human cerebral endothelium did not express CD73. Instead, we found CD73 expression at the border of the Virchow-Robin space, which may reflect CD73^+^ astrocyte endfeet at the glia limitans [45]. CD73 was also evident as punctate staining in HLA class II^+^ cells, most likely representing macrophages or microglia that have phagocytosed myelin, which is known to contain CD73 [40]. Collectively, the expression patterns of AP, CD39 and CD73 in MS lesions further underscore the importance of extracellular nucleotides, which were previously reported to drive leukocyte entry into the brain [24], mediate the suppressive action of Treg cells [43], and signal microglia to respond to tissue damage [46].

The production of AP *in vivo* is increased upon LPS challenge [47,48], and, also, IgM and IgG antibodies to the core domain of endotoxin are modulated by exposure to LPS [49]. To determine the need for AP supplementation in MS, we measured AP activity and determined endotoxin exposure by direct and indirect methods. We found no significant differences in AP activity and isoenzyme frequencies in MS patients compared to controls, although AP activity and AP isoenzyme distribution were slightly altered in PP-MS patients. Another study also addressed AP activity in RR-MS patients and reported comparable AP activity levels in MS versus healthy controls [50]. To our knowledge there are no other reports on AP activity in PP-MS patients. Parallel to an increase in AP levels in PP-MS, we detected a significant decrease of Endocab IgM levels in PP-MS patients compared to RR-MS patients and healthy controls, a finding that may be explained by differences in age as we found a significant negative correlation between age and Endocab IgM levels. In other human disease conditions, decreased Endocab IgM levels have been interpreted as consumption of antibodies caused by systemic release of gut endotoxin, for example, after cardiac surgery [49]. Similarly, low base-line levels of Endocab IgM and IgG are associated with increased mortality and prolonged hospitalization after surgery [51] and with the development of systemic inflammatory response syndrome (SIRS) in children with organ failure that occurred post-operatively or after head injury [52]. In contrast, elevated levels of IgG Endocab are found in Crohn’s disease, perhaps reflecting chronic exposure to endotoxin due to impaired gut barrier function [53]. Since the Endocab IgG levels in PP-MS patients were comparable to controls, it is unlikely that PP-MS patients have been chronically exposed to endotoxin, at least to the rough-type of LPS used in the Endocab assay.

## Conclusions

In conclusion, using the EAE model we found that administration of AP during the priming phase was effective in reducing clinical severity and proliferation of T cells in response to MOG35-55. We also demonstrated a strong expression of the AP-like enzyme CD39 in MS lesions, as a possible reflection of the microglial response to inflammation and tissue damage. Although the number of patients that were used to make comparisons and correlations in our study is modest and confirmation of the findings is required in an independent cohort, our results do suggest that MS patients may have sufficient circulating AP levels and neutralizing antibodies to inactivate and/or clear endotoxin. Nevertheless, upon microbial exposure or gut barrier dysfunction, when endotoxin is released into the systemic circulation, peripheral leukocytes could still be activated in a non-specific manner driving neuroinflammatory disease. The interrelationships between infections, microbial translocation of gut microbiota compounds and MS disease activity, therefore, warrants further investigation.

## Abbreviations

AP: Alkaline phosphatase; BIAP: Bovine intestinal alkaline phosphatase; BSA: Bovine serum albumin; CFA: Complete Freund’s adjuvant; CNS: Central nervous system; EAE: Experimental autoimmune encephalomyelitis; ELISA: Enzyme-linked immunosorbent assay; Endocab: Endotoxin core antibodies; Ig: Immunoglobulin; LAL: Limulus amebocyte lysate; LPS: Lipopolysaccharide; MOG: Myelin oligodendrocyte glycoprotein; MS: Multiple sclerosis; PBS: Phosphate buffered saline; PHA: Phytohemagglutinin; PP-MS: Primary progressive MS; RR-MS: Relapsingremitting MS; TNF-α: Tumor necrosis factor α; Treg: Regulatory T cells.

## Competing interests

The authors declare that they have no competing interest. Design, execution and interpretation of all experiments was performed by independent academic researchers without formal ties to the SME involved. RB gave advice on experimental design, provided pre-titrated AP and commented on the final draft of the manuscript.

## Authors’ contributions

RH and SO designed and performed animal experiments, immunohistochemistry and EndoCab assays. RH analyzed and interpreted the data and wrote the manuscript. KLK selected patients and controls for the study, performed endotoxin assays, analyzed data and commented on versions of the manuscript. JB carried out AP assays and participated in the interpretation of the serological results. RB and RQH gave advice on experimental design, commented on the final draft of the manuscript and provided pre-titrated AP (RB) or collected patient data (RQH). JDL conceived and designed the study, participated in its coordination and helped to draft the manuscript. All authors read and approved the final manuscript.
